# Biomarkers in Acute Kidney Injury

**DOI:** 10.4137/bmi.s450

**Published:** 2008-02-26

**Authors:** Jean-François Naud, Martine Leblanc

## Introduction

Acute Renal Failure (ARF) remains a common and significant problem in modern medicine. The epidemiology and causes of ARF vary according to the clinical setting; in intensive care units it is estimated that the incidence of ARF ranges from 10%–25%,^1^,^2^ with acute tubular necrosis (ATN) accounting for about 75% of cases.^3^

Recent data from the BEST kidney investigators has estimated the worldwide prevalence of ARF that is likely to require renal replacement therapy (RRT) amongst ICU patients to be as high as 5.7%, and is associated with a high hospital mortality rate.^4^ Despite recent major advances in the knowledge of the underlying mechanisms leading to kidney dysfunction, only small improvements have been made with respect to its prevention and treatment, and ARF continues to be a major contributor to in-hospital mortality.^5^,^6^

While past clinical trials in patients with ARF have been hampered by the lack of a standardized definition of ARF, recent meetings have resulted in validated stages of ARF as defined by the RIFLE criteria.^7^,^8^ These well-defined stages of ARF allow current trials to be standardized with respect to measures of kidney dysfunction, which is essential when evaluating patients for ARF. However, there still remains much improvements to be made in increasing the sensitivity of current markers of acute kidney injury (AKI). Creatinine is currently the most widely-used marker of renal function. Its use in the diagnosis of ARF remains a problem, however, as it often requires as much as a 50% loss in renal function before creatinine levels rise.^7^ The fact that the many different therapeutic agents that have been tried in ARF have shown very little success is perhaps explained by this delay in diagnosing ARF, which has been compared to beginning the treatment of acute myocardial infarction 48–72 hours after the coronary occlusion.^9^

For these reasons, there has been a search for better markers of early AKI in recent years, which have seen the advent of several promising new biomarkers of renal function in this setting. This review will discuss some of these promising biomarkers and their potential as useful markers of AKI. A literature search performed on MEDLINE/PubMed using the search terms ‘renal insufficiency, acute’ and ‘biological markers’ yielded 414 results, of which relevant articles were selected from all publication types in the English language, from either human or animal models.^10^

## Diagnosis of ARF

Current diagnosis of ARF relies on standard markers of renal function, and is usually accomplished through elevations in the levels of serum creatinine, urea, and urinalysis changes. Recently, RIFLE criteria have been described with regards to the use of these indices in the diagnosis of ARF.^7^ Briefly, ARF was defined as a spectrum initially starting with a risk of kidney injury (R criterion) with an increase in baseline creatinine ≥50% or urine output ≤0.5 mL/kg/h for 6 hours, to end-stage renal disease (E criterion) with anuria ≥3 months. Acute tubular necrosis (ATN), being the most common cause of ARF in hospitalized patients, is sometimes mistakenly used interchangeably with ARF. ATN, however, is a subset of ARF which results from ischemic or toxic injury to the renal tubular cells as opposed to other causes of ARF which include prerenal azotemia, urinary tract obstruction, vasculitis, glomerulonephritis, or interstitial nephritis. The distinction of ATN from these other causes of ARF has been reviewed in recent years and remains largely based on a combination of clinical history, physical examination and the standard laboratory studies mentioned above.^7^,^11^,^12^,^13^

Plasma creatinine, while being the most commonly used marker of renal function, is a suboptimal marker of ARF for several reasons. It is well known that factors such as liver dysfunction (leading to decreased creatinine production from creatine), low muscle mass, increased tubular secretion of creatinine in low-flow states, drug interference with tubular handling, and increased volume of distribution in critically ill patients frequently lead to an overestimation of renal function from serum creatinine measurements.^7^,^11^,^12^,^13^ Moran and Myers observed various opposed patterns of plasma creatinine change with regards to the measured glomerular filtration rate (GFR) in the non-steady state that represents ARF, demonstrating the inaccuracy of simple creatinine measurements in this setting.^14^

Blood urea nitrogen (BUN) determination has also been used extensively in the evaluation of kidney function. Levels of BUN also present several confounding factors as they depend on exogenous urea load, endogenous production, and tubular reabsorption, which is increased in states of reduced effective renal perfusion.^15^ Like creatinine, its use in the diagnosis of ARF has therefore not been without limitations.

## Renal Tubular Cell Proteins and Enzymes

### N-acetyl-β-D-Glucosaminidase and tubular brush-border enzymes

Several enzymes originating from proximal tubular cells have been investigated as markers of necrotic damage whose urinary levels are increased following proximal tubule injury or dysfunction. Their use as markers of proximal tubular injury has been reviewed recently.^16^ The acute or chronic damage of the renal tubular cells is believed to cause the release of these enzymes from the renal tubules into the urine where they can be measured. Many urinary enzymes were studied in the last 25 years, originating broadly from the lysosomes, the brush-border membrane and the cytoplasm of the renal tubular cells. N-acetyl-β-D-Glucosaminidase (NAG), found predominantly in proximal tubule lysosomes, has been the most extensively studied of these enzymes in ARF in various contexts including nephrotoxic agents, ATN, after cardiac surgery or renal transplantation.^17^–^23^ Other enzymes that have been investigated include alkaline phosphatase (AP), alanine amino-peptidase and γ-glutamyl transpeptidase (GGT), which are enzymes found in the brush-border membrane, while the different isoforms of glutathione-S-tranferase (α and γ GST) are cytosolic enzymes.^24^ The measurement of such enzymes is currently done by automated assays usually via immunonephelometric methods or ELISA.

However, despite the fact that enzymuria clearly reflects tubular injury, the clinical significance of enzymuria regarding early diagnosis of ARF has been questionned as it may not always imply progression towards clinical renal failure.^23^,^25^ Likewise, it has been shown on several occasions that contrast administration during coronary angiography or arteriography provoked an elevation in urinary NAG, however again without necessarily progressing towards clinically apparent renal failure.^26^–^28^ Similarly, NAG has been known to be elevated in other types of kidney diseases, such as diabetic nephropathy, perhaps limiting its usefulness in this setting.^29^

In a small study involving 26 ICU patients of whom 4 developed ARF (defined as a creatinine increase ≥50% or ≥0.15 mmol/L) Westhuyzen et al. evaluated urinary NAG, GGT, AP, α/γ GST, and lactate dehydrogenase (LDH) values at ICU admission in the early detection of ARF.^30^ Receiver-operating curve (ROC) analysis for GGT, α and γ-GST, AP and NAG was performed despite the low number of events (area under curve of 0.95, 0.92, 0.89, 0.86 and 0.84 respectively), while the ROC analysis for LDH and creatinine clearance was lower albeit still significant. Plasma creatinine detected the onset of ARF between 12–96 hours after admission, and BUN determination was unhelpful in diagnosing ARF. Tubular enzymuria at admission in the ICU was thus useful in their small ICU sample in predicting the development of ARF, and more so in ruling out the development of ARF.

A known limitation of enzymuria, however, concerns the inability of these enzymes to help differentiate between the different clinical causes of ARF. Chew et al. were unable to differentiate between the various etiologies of ARF using NAG, intestinal-type AP and tissue non-specific alkaline phosphatase, in 50 patients with ARF (n = 16 for prerenal ARF, n = 28 for renal ARF, and n = 6 for post-renal ARF).^18^ Similarly (see KIM-1 study below), Han et al. could not differentiate between the causes of ARF in their cohort of 32 patients with ARF using GGT and AP.^31^

In an interesting study, Herget-Rosenthal evaluated the usefulness of tubular proteinuria and enzymuria for predicting the need for renal replacement therapy (RRT) in 73 patients who developed non-oliguric ARF due to ATN, of whom 26 required RRT.^32^ All patients with established non-oliguric ARF fulfilling ATN criteria were included in the study and had their urine collected on the day of inclusion for levels of α-1 and β-2 microglobulins, cystatin C, retinol-binding protein, α-GST, GGT, LDH, and NAG. Patients requiring RRT had significantly increased NAG values as opposed to patients who did not, and the area under the ROC curve was 0.81 for urinary NAG values above 4.5 U/mmol of urinary creatinine. However the sensitivity/specificity of urinary NAG for RRT was lower than that of cystatin C and α-1-microglobulin, being 85%/62%. Patients who required RRT also had significantly higher urinary values of cystatin C and α-1-microglobulin than patients who did not require RRT. The other markers evaluated (retinol-binding protein, β-2-microglobulin, GST, GGT, LDH) showed less ability to predict the need for RRT.

Liangos et al. obtained concordant results lately in a study that evaluated both NAG and KIM-1 (see below for KIM-1). Higher levels of both NAG and KIM-1 were associated with higher odds ratio for dialysis requirement or hospital death in a larger cohort of 201 patients separated in four different quartiles according to NAG values.^33^ Of note, there were no statistical differences in creatinine values between the 4 different quartiles, despite differences in the proportion of patients requiring RRT. Unfortunately, the NAG values for the four different quartiles were not reported.

Overall, these studies indicate that quantifying enzymuria remains a sensitive, albeit not very specific, tool for establishing the presence of a renal tubular injury. Recent studies have suggested a promising role for NAG in helping to predict adverse outcomes in ARF, and future studies should try to validate these results in larger cohorts.

### Kidney injury molecule-1 (KIM-1)

KIM-1 was originally described as a putative epithelial cell adhesion molecule upregulated in rat renal proximal tubules after renal ischemic injury by Ichimura et al. in 1998.^34^ While its role remains unclear, it consists in a transmembrane protein whose ectodomain is shed into the urine where it can be measured. In a subsequent study involving 32 patients with various forms of acute and chronic renal disease published in 2002, urinary KIM-1 levels were shown to be significantly more elevated in patients with ATN as opposed to patients with other causes of ARF or chronic renal disease.^31^ The 7 patients with ischemic ATN had mean normalized urinary KIM-1 values of 2.92 +/− 0.61, while the remaining patients with ARF had mean values of 0.63 +/− 0.17, p ≤ 0.01 (16 total patients, 7 with contrast nephropathy, 5 with prerenal azotemia, 2 with cyclosporine toxicity, 1 with post-obstructive nephropathy and 1 with interstitial nephritis). Patients with chronic renal diseases had mean normalized values of 0.72 +/− 0.37, p ≤ 0.01 (n = 9, diseases included Wegener’s granulomatosis, systemic lupus erythematosus nephropathy, diabetic nephropathy, focal segmental glomerulosclerosis, and chronic allograft dysfunction). Of note, the definition of ARF used in the study was 1) increase in serum creatinine ≥0.5 mg/dL if baseline ≤2.0 mg/dL, or 2) increase in serum creatinine ≥1.5 mg/dL if baseline ≥2.0 mg/dL, or 3) increase in serum creatinine ≥0.5 mg/dL regardless of baseline if as a consequence of exposure to contrast agents. Urinary AP and GGT were also measured but were not helpful in differentiating ATN from other causes of renal injury.

Since then, urinary KIM-1 levels have been shown to be increased in rodents receiving nephrotoxic doses of cisplatin, folic acid, cadmium, gentamycin, mercury and chromium,^35^–^39^ suggesting a role of KIM-1 for detecting nephrotoxic insults. Also, Liangos et al. demonstrated very recently in a cohort of 201 patients with ARF separated in quartiles according to their urinary levels of KIM-1, that higher levels correlated with a higher odds ratio for dialysis requirement or hospital death, adding some prognostic value to the measured levels of urinary KIM-1.^33^ As with NAG values however, the actual values of the different KIM-1 by quartiles were not reported. These results all suggest a useful role of KIM-1 in detecting renal ischemic injuries, but further studies from larger cohorts will be required to validate this biomarker in current practice.

### Na^+^/H^+^ exchanger isoform 3 (NHE3)

The NHE3 is the most abundant of all recently detected sodium transporters expressed in the nephron and can be detected in urine.^40^ It is localized in the apical membrane and subapical endosomes of renal proximal tubular cells and in the apical membrane of thick ascending limb cells,^41^–^43^ and is responsible for 60%–70% of the reabsorption of the filtered sodium.^44^ Du Cheyron et al. measured the urinary levels of NHE3 in 68 patients admitted to the ICU, 54 of whom either had or developed ARF during their ICU stay (defined as an increase in serum creatinine to ≥177 μmol/L or to a value ≥50% of basal creatinine when chronic renal insufficiency existed).^45^ Patients with ARF were divided into 3 subgroups according to clinical criteria: prerenal azotemia (which improved rapidly after volume replacement), ATN (defined as renal dysfunction that did not improve after correction of possible prerenal causes and could not be attributed to other causes), and intrinsic ARF other than ATN. In this population, levels of urinary NHE3 normalized for urine creatinine were increased six times as much in patients with ATN than in those with prerenal azotemia (0.78 +/− 0.36 vs. 0.12 +/− 0.08, p ≤ 0.001), while urinary NHE3 could not be detected in the 14 control ICU patients without ARF. Urinary RBP was also measured but could not discriminate between prerenal ARF and ATN.

### Urinary actin

Kwon et al. evaluated urinary actin in a study that also looked at interleukin-6 (IL-6), interleukin-8 (IL-8), GGT, LDH, and tumor necrosis factor-alpha (TNFα) in 40 kidney transplant recipients during the first post-transplant week.^46^ Actin is the main cytoskeletal protein in cells, and damage to the cellular cytoskeleton has been described as one of the key events following renal ischemia-reperfusion injury.^47^,^48^ Of these 40 patients, 9 out of the 30 patients receiving cadaveric allografts progressed to sustained ARF (defined as a creatinine clearance ≤25 mL/min by 24 hour urine collection at day 7 post-operatively), while the remaining 21 went on to a recovery phase (defined as a creatinine clearance above 25 mL/min). All 10 patients receiving living donor allografts progressed to a recovery phase. In their study urinary actin at day 0 was elevated in recipients destined to have sustained ARF, with ROC analyses for urinary actin at day 0 yielded values of 0.75. This suggests that urinary actin might serve as a predictor of sustained ARF after kidney transplantation from cadaveric allografts, but larger studies will be required to further explore the role of urinary actin in AKI.

### Exosomal fetuin A

All nephron segments secrete exosomes containing apical membrane and intracellular fluid into the urine under normal conditions and thus may potentially carry protein markers of structural damage to the nephrons. Exosomal fetuin A, an acute phase protein involved in various inflammatory states, was very recently identified using urinary proteomics techniques from a rat models of cisplatin-induced nephrotoxicity and ischemic-reperfusion injury as a potential marker of AKI in these settings.^49^ Urinary exosomal fetuin A was then also shown in the same study to be elevated in 3 ICU patients with AKI compared to the patients without AKI. Fetuin A might thus be a potential urinary biomarker of AKI, and will need to be investigated in larger studies.

### Cysteine-rich protein 61 (Cyr61)

Cyr61 is a secreted heparin-binding protein that may possibly be involved in tissue growth and repair,^50^,^51^ which was originally described as a growth factor-inducible immediate early gene in fibroblasts.^52^ It was identified by Muramatsu et al. in 2002 by representational difference analysis as one of the genes that was rapidly induced following ischemia in rodent models of ischemic/reperfusion injury.^53^ The urinary levels of Cyr61 in rodents could be detected as early as three to six hours following renal injury, where it might eventually serve as an early biomarker of ARF. More studies will be required to validate its use in humans.

### Sulfated HNK-1 epitope

The HNK-1 carbohydrate epitope is attached to lactosamine structures on glycoproteins, proteoglycans, or glycolipids and has been known to be an important actor in neurogenesis and immune systems^54^,^55^ through proposed involvement in cell-cell interactions and cell migration.^56^ Such interactions are likely to be involved in renal morphogenesis, as Allory et al. demonstrated in 2006 the expression of the HNK-1 epitope mainly to the thin ascending loop of Henle in adult kidneys.^57^ Furthermore, in 10 kidney biopsies from patients with ATN after renal transplantation, the HNK-1 epitope was found to be expressed at various levels in 8 of these cases, thus raising the possibility of its use as a potential marker for ATN.

## Urinary Low-Molecular Weight Proteins

Urinary excretion of low-molecular weight (LMW) proteins, like that of renal proximal tubular enzymes, has also been extensively described in the biomarker literature over the last 25 years. These LMW proteins are usually freely filtered and then reabsorbed by the proximal tubule under normal conditions.^58^ In settings of tubular damage where such reabsorption is impaired, or in contexts of increased reabsorptive load with increased transglomerular passage of proteins, these proteins cannot be entirely reabsorbed and are therefore secreted in urine.^59^ These proteins that have been used as biomarkers of tubular dysfunction include β-2-microglobulin, α-1-microglobulin, retinol-binding protein (RBP), and cystatin C.

In a study from 2004 described previously, Herget-Rosenthal evaluated the usefulness of tubular proteinuria and enzymuria for predicting the need for RRT in 73 patients who developed non-oliguric ARF due to ATN, of whom 26 required RRT.^32^ Patients who required RRT had significantly higher urinary values of cystatin C and α-1-microglobulin than patients who did not require RRT. The area under the ROC curves for these two proteins was 0.92 and 0.86 respectively, and with a sensitivity/specificity of 92%/83% and 88%/81% respectively, at values of urinary cystatin C ≥1g/mol of creatinine and α-1-microglobulin ≥20g/mol of creatinine. As said previously, patients requiring RRT also had significantly increased NAG values as opposed to patients who did not, however the sensitivity/specificity of urinary NAG for RRT was lower than that of cystatin C and α-1-microglobulin. The other markers evaluated (RBP, β-2-microglobulin, GST, GGT, LDH) showed less ability to predict the need for RRT.

While these results appear promising for cystatin C and α-1-microglobulin, previous studies involving the latter had shown that while being a sensitive marker of tubular injury, elevations in α-1-microglobulin were not always associated with clinically relevant renal injury,^60^–^62^ which is similar to the use of several renal biomarkers described previously in this review.

### Cystatin C

Cystatin C is a low molecular weight protein functioning as a lysosomal cysteine protease inhibitor, and is therefore strongly implicated in the regulation of proteolytic damage of these enzymes.^63^ Cystatin C is considered one of the housekeeping genes and is produced at a constant rate by all nucleated cells, without changes with aging, gender, or muscular mass. By virtue of its low molecular weight, it is freely filtered at the glomerulus and is completely catabolized by proximal tubules.^64^–^66^ These characteristics thus make cystatin C a good marker of glomerular filtration rate whose reliability is comparable or superior to plasma creatinine, and has been demonstrated in several clinical settings.^65^,^67^–^72^ In ARF, however, there exist only a few clinical studies which evaluated cystatin C. Hergert-Rosenthal demonstrated the clinical significance of cystatin C in ARF.^73^ In this study, serum cystatin C and serum creatinine were measured daily in 85 critically ill patients at high risk of developing ARF. In the 44 patients with ARF (classified according to the recent RIFLE criteria^7^), a significant early rise in cystatin C detected a risk of renal injury (R criteria, elevation of ≥50% from baseline creatinine) 1.5 +/− 0.6 days before a rise in serum creatinine. Applying the same analysis to renal injury and renal insufficiency (I and F criteria) also detected these conditions earlier in a similar fashion. Regarding the need for RRT, an elevation of cystatin C ≥50% predicted RRT requirements moderately well, with a sensitivity of 53% and specificity of 82%. Herget-Rosenthal also demonstrated the use of urinary cystatin C in 2004 in predicting the need for RRT in patients with non-oliguric ARF due to ATN, as just described.^32^

Also, Ahlström et al. measured serum cystatin C daily starting from admission to the ICU in 202 patients.^74^ ARF (defined here as a *threefold* increase in baseline creatinine, or in case of chronic renal failure as an increase ≥0.5 to 4.0 mg/dL of creatinine, or diuresis ≤0.3 mL/kg/h for 24 h, or the need for RRT) occurred in 54 patients, and cystatin C was as useful as creatinine in detecting ARF. Plasma creatinine, however, was already at a mean of 255 μmol/L at ICU admission in the group of patients that developed ARF (with mean peak values of plasma creatinine of 294 μmol/L), and RRT was started on day 2 on average, suggesting pre-existing advanced renal dysfunction at the time of study inclusion. When only considering abnormal values of creatinine that developed after ICU admission (defined in the protocol as ≥90 or 95 μmol/L for females and males, respectively), both serum cystatin C and plasma creatinine appeared equally quickly (median 3 days). It is unspecified if any of those 29 patients went on to develop ARF, however.

Of note, one small study has produced conflicting results regarding the use of cystatin C in ARF. It involved 29 critically ill septic patients of which 10 developed ARF (defined here as creatinine ≥267 μmol/L or diuresis ≤30 mL/h, duration not specified), cystatin C was not found to be correlated with the occurrence of ARF, as opposed to measurements of the N-terminal prohormone of atrial natriuretic peptide (proANP).^75^ It is unclear from this study how many patients required RRT.

Still, another small study from Delanaye et al. showed that the ability of cystatin C to detect a GFR ≤ 80 cc/min/1.73m^2^ (GFR calculated by Cockroft-Gault estimation or by 24h creatinine clearance) was superior than plasma creatinine in 14 ICU patients.^76^ Similarly, a report from ten patients undergoing unilateral nephrectomy for transplantation purposes also demonstrated that serum cystatin C increased by 50 to 100% postoperatively 1.4 +/− 0.9 days earlier than creatinine (p = 0.009).^77^

Finally, in 2006, a study by Zhu et al. demonstrated the ability of serum cystatin C in detecting acute renal dysfunction (defined as a creatinine clearance below 80 mL/min/1.73m^2^ measured by 24 h urine collection) in 60 patients undergoing heart valve replacement.^78^ Cystatin C levels peaked at post-operative day 2 on average as opposed to day 3 for creatinine, and cystatin C levels significantly rose in 19 of the 26 patients developing acute renal dysfunction while only 7 of these patients demonstrated an elevated serum creatinine. Interestingly, ‘low-dose’ corticosteroid therapy (dexamethasone 10 mg daily for 3 days after surgery, n = 26) did not result in any changes in measured serum cystatin C when compared to patients not receiving any corticosteroids. Increased serum cystatin C levels have been reported in patients receiving corticosteroids,^79^–^82^ but other investigators have reported so only after large, prolonged doses.^80^

It should also be noted that in settings of radio-contrast nephropathies, an evaluation of the nephrotoxic effect of contrast administered during coronary angiography demonstrated a significant elevation of cystatin C (+7.2%) 24 hours after angiography, while the serum creatinine rise could only be seen 48 hours after, also suggesting a role of cystatin C in the diagnosis of AKI.^83^ Likewise, in a study by Bachorzewska-Gajewska evaluating NGAL in coronary angiographies, the levels of serum cystatin C were also significantly elevated 24 hours after coronary angiography (from 1.69 +/−1.03 to 2.85 +/−2.05 mg/L, p < 0.01), while serum creatinine remained unaffected.^84^

Cystatin C is a well-established marker of GFR in settings of stable renal function and chronic renal insufficiency.^65^–^72^ Taken together, the results of the studies just described also seem to suggest a possible role for serum cystatin C in detecting AKI, and perhaps especially for urinary cystatin C, also in predicting adverse outcomes in ARF such as RRT. In view of the several conflicting studies, it seems likely however that larger studies will be required to clarify the role of cystatin C in ARF.

### Neutrophil gelatinase-associated lipocalin (NGAL)

NGAL is a part of the lipocalin protein family and is a 23 kDa low molecular weight protein secreted by various types of human cells, which include not only activated neutrophils^85^ but also other tissues such as the kidneys, and cells of the gastro-intestinal and respiratory tracts.^86^–^91^ NGAL is also strongly expressed by various types of carcinomas and adenomas. Its physiologic role seems complex, implying cell growth and differentiation, a bacteriostatic immune effect and a role in cellular iron-transport pathways.^92^ By virtue of its small size NGAL is freely filtered by the renal glomeruli without being reabsorbed, and can therefore be measured in the urine. Early studies by Mishra et al. in 2003 using cDNA microarray assay methods allowed its identification as a protein strongly expressed in renal tubules in animal models of renal ischemic injury.^93^ Interestingly, NGAL might act in a protective fashion for the renal tubules in this context, as seem to indicate some animal studies.^94^

In humans, the role of this new biomarker in settings of AKI has now been demonstrated. In a recent study by Mishra et al. urinary and plasma NGAL levels were measured in 71 children undergoing cardiac surgery necessitating extra-corporeal cardio-pulmonary bypass.^95^ Amongst the 20 children developing AKI (defined as an elevation of serum creatinine >50% from baseline), a significant rise in the average urinary NGAL levels was seen as early as 2 hours post-operatively (from 1.6 μg/L SE 0.3, to 147 μg/L SE not available), while a significant change in serum creatinine could only be noted from 24 to 72 hours postoperatively. Using a urinary NGAL cutoff value of 50 μg/L and looking at the 51 children not developing AKI as case-controls, the urinary NGAL level 2 hours after CPB showed an impressive 100% sensitivity and 98% specificity in the early diagnosis of AKI. Plasma NGAL levels showed similar results although sensitivity and specificity of the test were somewhat lower than for urinary NGAL in the study. A similar study in adults after cardiac surgery also demonstrated a significant increase in urinary NGAL 1 hour after surgery, however without being adequately powered to obtain sensitivity and specificity values.^96^

Interestingly, Bachorzewska-Gajewska recently demonstrated a rise in urinary NGAL 4 hours following the administration of contrast during percutaneous coronary angioplasties in 25 adult patients (from 11.1 μg/L +/−15.8 to 17.8 +/− 34.48, p < 0.05) despite however any significant change in average serum creatinine values.^84^ These studies thus suggest a very promising role for NGAL in the early evaluation of ARF which will require further investigations.

### Urinary interleukin-18 (IL-18) and other cytokines

IL-18 is a cytokine with several important roles in human immune defense mechanisms, principally acting as a co-stimulator in the production of gamma-interferon, a crucial element in the human defenses against infections. At the renal level, recent animal studies in mice by Melnikov demonstrated the possible role played by IL-18 in the renal tubules in settings of acute ischemic tubular necrosis.^97^,^98^

It was then demonstrated in humans that the urinary concentration of IL-18 was higher in ATN when compared with other causes of renal insufficiency such as hypovolemia, heart failure, urinary tract infections and chronic renal failure (n = 72).^99^ In the same study, the urinary IL-18 level at 24 hours after kidney graft was also much higher in patients who later developed delayed graft dysfunction. The same investigators also looked at a cohort of 72 children undergoing cardiac surgery requiring cardio-pulmonary bypass.^100^ They demonstrated a significant increase in the urinary IL-18 level starting only 4 hours after surgery in the 20 children who developed an acute renal injury (as defined by an increase >50% of baseline creatinine in the 3 days after surgery) when compared to 35 case-controls chosen amongst the 50 children without renal insufficiency. The sensitivity and specificity of IL-18 in this context seemed best between 12 and 24 hours with values of 40%–50% and 94% respectively, for urinary IL-18 levels higher than 50 pg/mL. The area under the ROC was 0.73 and 0.75 for these levels of IL-18 at 12 and 24 hours. One other case-control study with urinary samples from 52 cases and 86 control-cases (controlled for demographic factors, sepsis, baseline creatinine, APACHE III score, and diuresis) involved in the ARDS Network Study demonstrated a relationship between a rise >100 ng/mL of IL-18 at 24 and 48 hours and the development of ARF.^101^ This rise in urinary IL-18 was also associated with higher mortality amongst these patients.

These studies thus indicate a possible role for urinary IL-18 in the early evaluation of ARF, and also an ability to differentiate between some of the causes of ARF. Once again, more studies will be needed to validate these early results.

Interleukin-6, interleukin-8, and tumor necrosis factor-alpha are other inflammatory cytokines that have been implicated in the cascade of cellular events that follows renal ischemic injury.^102^–^104^ Their role in the detection of AKI was investigated in a study by Kwon et al. (mentioned previously) in the context of early kidney transplant graft rejection.^46^ In their study, IL-6 and IL-8 (but not TNFα) at day 0 were elevated in recipients destined to have sustained ARF. ROC analyses for urinary IL-6, and IL-8 at day 0 yielded values of 0.91, and 0.82 respectively. Such results thus suggest that these markers might serve as strong predictors of sustained ARF after kidney transplantation, but larger studies should obviously be done to ascertain these early results.

## The Future Search for Biomarkers

As several recent reviews have mentioned, the recent advent of several new biomarkers, each representing a different aspect of the various causes of ARF (see Table 1), is likely to continue as our understanding of the different molecular mechanisms involved in ARF evolves.^105^,^106^ Through genomics and proteomics, recent studies have looked at the different genes and proteins expressed during different models of ARF and identified several other candidate biomarkers.^107^ Several studies using cDNA microarray methods have already identified many genes that are either upregulated or downregulated in animal models of ARF.^108^,^113^–^115^ A recent study also demonstrated the differential gene expression between different models of ARF that included nephrotoxic injury, ischemic injury and hypovolemia.^108^ As seen in this study however, a frequent problem with genomic approaches is that modifications in gene transcription do not always result in altered protein expression because of various post-transcriptional events. Changes at the genomic level therefore also need to be evaluated in terms of changes in functional protein levels. Another exciting approach has been through the recent development of the field of proteomics, and particularly of the urinary proteome.^109^ This approach focuses on the pattern of urinary protein expression using mass spectrometry to identify the proteins implicated with the cause of renal disease. In recent years, proteomics has proven to be a powerful tool in investigation and clinical medicine, and will likely eventually provide us with a comprehensive knowledge of the various proteins that can be found in the urine under many conditions.^109^–^112^ An important problem with proteomics is however to ensure the stability of the proteome from the collection to analysis for there are several factors that might alter samples.^112^ Also the functional role of the various proteins identified might not necessarily be directly proportional to their amount, and the identification of scarce compounds requires methods with high sensitivity which can prove to be difficult. Finally, standards will have to be established for means of comparison. Still, the research potential of these methods is obviously enormous, and one should expect large amounts of data to come forward from these in the next years as our knowledge and techniques evolve.

There are therefore many different biomarkers of kidney function that each reflect different aspects of renal physiology, and which are all affected differently in the various conditions that result in ARF. Genomics and proteomics have already yielded extremely interesting insights in our understanding of the pathophysiology of the various forms of ARF and their associated potential biomarkers, and will undoubtebly continue to do so in the next few years. It is likely that no single marker will prove to have the required sensitivity and specificity required for all these different diagnoses, and we will most likely depend on a panel of some of the markers implicated in the various forms of ARF in its different phases to properly diagnose ARF in the future.

## Figures and Tables

**Table 1:** New biomarkers of acute kidney injury (see text).

Biomarker	Uses in humans	Type of assay
Renal tubular cell proteins		
KIM-1	ATN	ELISA
NHE3	ATN	Immunoblot
Actin	Delayed graft function	Western Blot
Cyr61	ATN	Western Blot
Urinary low-molecular weight proteins		
Cystatin C	AKI	ELISA
NGAL	AKI	ELISA
IL-18	ATN, AKI	ELISA
IL-6	Delayed graft function	ELISA
IL-8	Delayed graft function	ELISA
